# The association between interest of nutritional supplements and COVID-19 pandemic - evidence from Google Trends

**DOI:** 10.1186/s12889-023-17607-2

**Published:** 2024-01-06

**Authors:** Li-Yin Lin, Atina Husnayain, Yi-Tui Chen, Chao-Yang Kuo

**Affiliations:** 1 Department of Leisure Industry and Health Promotion, National Taipei University of Nursing and Health Sciences, Taipei, 112 Taiwan; 2 Department of Public Health, Monash University, Banten, 15345 Indonesia; 3 Smart Healthcare Interdisciplinary College, National Taipei University of Nursing and Health Sciences, Taipei, 112 Taiwan; 4 Department of Education and Research, Taipei City Hospital, Taipei, 103 Taiwan

**Keywords:** Google Trends, Nutritional supplements, Relative search volumes, COVID-19

## Abstract

**Background:**

Due to the spread of the coronavirus disease 2019 (COVID-19) pandemic in 2020, the interest of nutritional supplements has emerged. Limited data are available on how the COVID-19 pandemic affects the search interest in nutritional supplements in Taiwan and worldwide. The study aims to investigate changes in public search interest of nutritional supplements pre- and during the COVID-19 pandemic.

**Methods:**

Our World in Data dataset was used to collect both global and local (Taiwan) number of COVID-19 newly confirmed cases and deaths. Google Trends search query was being used to obtain relative search volumes (RSVs) covering a timeframe between 2019 to 2022. Spearman’s rank-order correlation coefficients were used to measure relationships between confirmed new cases and deaths and RSVs of nutritional supplements. Multivariate analysis was conducted to examine the effect of domestic and global new cases and deaths on the RSVs of nutritional supplements.

**Results:**

The mean RSVs for nutritional supplements were higher during the COVID-19 pandemic period (between 2020 to 2022) compared to the pre-pandemic period (year of 2019) for both Taiwan and worldwide. In terms of seasonal variations, except for vitamin D, the mean RSVs of probiotics, vitamin B complex, and vitamin C in winter were significantly lower compared to other seasons in Taiwan. The RSVs of nutritional supplements were not only affected by domestic cases and deaths but also by global new cases and deaths.

**Conclusions:**

The interests in nutritional supplements had substantially increased in response to the COVID-19 pandemic. The RSVs of nutritional supplements in Taiwan were not only influenced by global and domestic pandemic severity but also by seasons.

**Supplementary Information:**

The online version contains supplementary material available at 10.1186/s12889-023-17607-2.

## Introduction

**Fig. 1:**
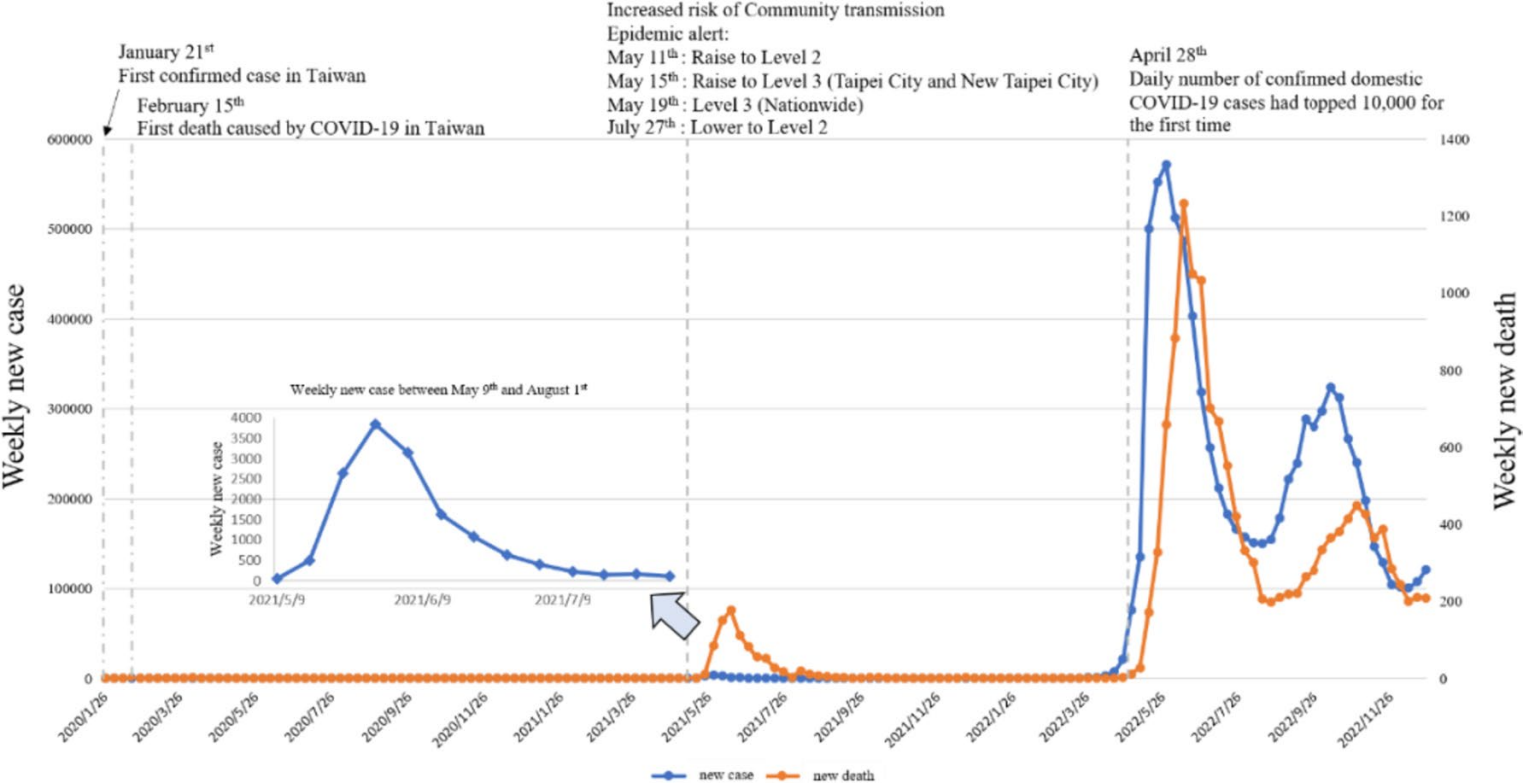
Weekly new cases and deaths in Taiwan between 2020 and 2022

 The COVID-19 outbreak, first reported on January 13, 2020, in Wuhan, China has become a serious global public health problem. The disease was caused by the novel severe acute respiratory syndrome coronavirus 2 (SARS-CoV-2) and has infected over 650 million people worldwide as of January 1, 2023 [1]. As for Taiwan, the Central Epidemic Command Center (CECC) reported the first case of COVID-19 on January 21st, 2020, followed by the first death associated with COVID-19 on February 15th, 2020. The first COVID-19 community transmission occurred in May 2021, and CECC raised a nationwide epidemic warning to Level 3 on May 19th, 2021. On April 28th, 2022, daily number of confirmed domestic COVID-19 cases exceeded 10,000 for the first time. Figure 1 illustrates the weekly new cases and deaths from January 21st, 2020 to December 25th, 2022.

COVID-19 affects the immune system by producing a systemic inflammatory response [2]. Common initial signs and symptoms include cough, fever, headache, fatigue, body aches, and diarrhea [3]. Some individuals with COVID-19 become severely ill, usually starting about 1 week after symptom onset; severe COVID-19 often involves progressive respiratory failure and may result in life-threatening pneumonia, multiorgan failure, and eventually death [2, 3]. Although COVID-19 is known to affect people across all lifespans, it has been shown that the most serious consequences occur in individuals with older age, individuals with chronic diseases, and those with impaired immune systems [4, 5].

Currently, there is a lack of data to support recommendations for or against the use of any vitamin, mineral, herb, fatty acid, or other dietary supplements to prevent or treat COVID-19. However, nutritional influence on the immune system has been well documented in the literature [6]. In general, where practicable, an efficient way to reduce the risk of viral infections is to regulate the actions of the inflammation mediators through adaptable risk factors such as diet and exercise [7]. As widely demonstrated in the literature, the intake of different nutritional supplements such as essential fatty acids, linoleic acids, essential amino acids, and vitamins and minerals can improve the immune response, especially where immunity can also be conditioned by nutrition deficiencies as in the presence of viral infections [8].

Nevertheless, dietary supplement sales have dramatically risen during the COVID-19 pandemic despite depressed economic conditions [9, 10]. Many people hoped that these products might provide some protection against SARS-CoV-2 infection and, for those who develop COVID-19, help reduce symptoms and disease severity [11, 12]. There are many vitamins and minerals necessary for the normal functioning of the immune system [13]. Commonly, the use of immune-modulating dietary supplements including vitamin B, vitamin D, ascorbic acid (Vitamin C), zinc, omega-3 fatty acids, and probiotics [14] has shown to have a positive effect on strengthening immunity [13, 15]. Additionally, the use of nutritional supplement is also influenced by season. For instance, the use of vitamin C and D nutritional supplements often increases during cold and flu seasons (typically known as autumn and winter seasons) [16, 17].

Vitamin B complex does not only build and maintain a healthy immune system but also potentially prevent or reduce COVID-19 symptoms [18, 19]. A study conducted on 9,189 adults aged between 20 to 69 years indicated higher intake of vitamin B5 could reduce the odds of COVID-19 by 47%, and a moderate intake of vitamin B12 had a protective effect on COVID-19 [19]. Vitamin C is well known for its antiviral properties, such as increasing interferon-alpha production, modulating cytokines, reducing inflammation, and restoring mitochondrial function [20]. Several studies have also shown that vitamin C reduces the risk associated with upper respiratory tract infections [20–22]. Vitamin D is known for its anti-inflammatory and antioxidant properties [23]. Vitamin D is also involved in the modulation of the immune response in infectious and autoimmune diseases [24]. A number of studies suggested that vitamin D inhibits the overexpression of inflammatory cytokines [25]. Probiotics modulate innate and adaptive immune responses, facilitating the immune system’s development and maturation. Therefore, the use of probiotics with anti-inflammatory effects could maintain the equilibrium of intestinal microecology and prevent secondary infection in COVID-19 [26].

Since nutritional supplements use appears to be increased in response to COVID-19 pandemic severity worldwide, the association between number of newly confirmed cases and deaths and nutritional supplement search interest becomes an interesting topic for further investigation. Previous studies had indicated both an increasing number of COVID-19 confirmed cases and deaths and the presence of COVID-19 outbreak led to an increased online search of immune-boosting related dietary supplements. Globally, the public believes the use of nutrients and herbs can improve self immunity to fight against COVID-19 infection, as well as improving overall health and wellbeing [12, 27]. Our World in Data Coronavirus Pandemic (COVID-19 dataset) provides a public aggregated global dataset on COVID-19 newly confirmed cases and deaths. It is widely used by journalists, policymakers, researchers and the public [28]. This dataset on the conronavirus pandemic is updated daily, publicly accessible, and allows users to select a specific country and tracks the newly confirmed cases and deaths. Both global and local (Taiwan) data on daily newly confirmed cases and deaths can be collected from Our World in Data dataset.

Google Trends is a search trend feature that shows how frequently a given search term is entered into Google’s search engine relative to the site’s total search volume over a given period of time. The benefits of Google Trends include showing information trends, networking on the Internet, and changes that can have a negative or positive effect on public health. Nowadays, Google Trends has been used in many studies to analyze the public’s search behavior [29]. Analyses of searches related to COVID-19 and nutritional supplements can reveal which nutrients are of the greatest interest and how that interest changes over time. Up to date, only a few studies have looked at Google queries about dietary supplements [27, 30]. In this study, we aim to compare the public interest in nutritional supplements pre- COVID-19 pandemic and during the COVID-19 pandemic (pre-COVID-19 pandemic: 2019; during the COVID-19 pandemic: 2020 to 2022) in Taiwan and worldwide.

## Materials and methods

### Study design

**Fig. 2:**
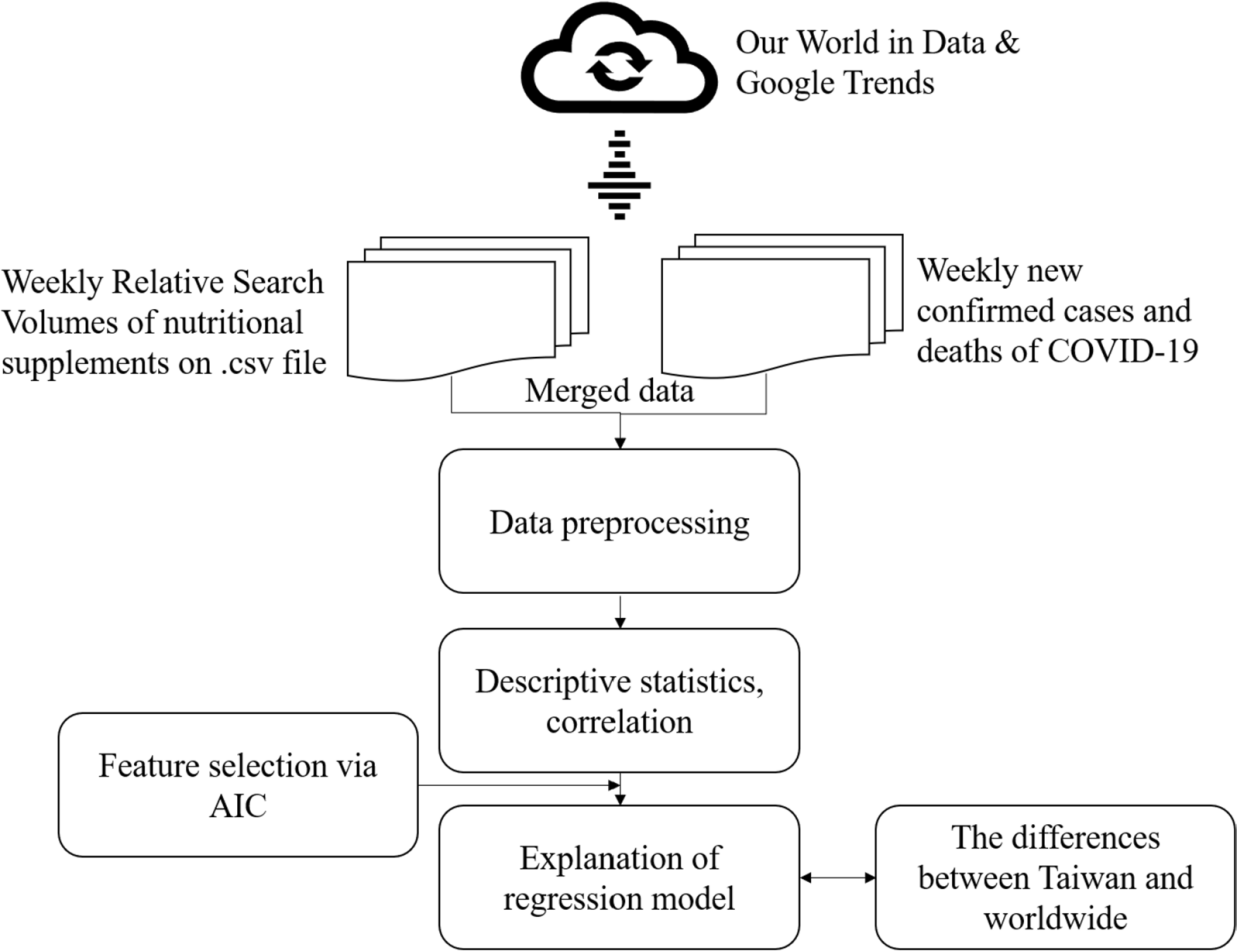
Experimental flowchart of study design

This is a quantitative research with the use of secondary dataset. We downloaded weekly new confirmed cases and deaths of COVID-19 and Relative Search Volumes (RSVs) of interested nutrition from “Our World in Data” and “Google Trends”, respectively. Descriptive statistics and correlation analysis were performed. Feature selection via the minimum Akaike’s Information Criterion (AIC), which is used to examine the goodness-of-fit of model with different groups of variables, was performed before regression analysis. Finally, the differences of interest in nutritional supplements between Taiwan and worldwide were being compared. The analytical flowchart is shown in Fig. 2.

### Data acquisition

Daily confirmed COVID-19 cases and deaths were retrieved from the “Our World in Data” website (https://ourworldindata.org/). The data collected by Our World in Data contains both local (Taiwan) and global data. A total of 156 weeks of COVID-19 confirmed case and death data was retrieved. RSVs of this study were obtained from Google Trends’ website. The COVID-19 pre-pandemic period was defined as the year of 2019, while the during pandemic period was defined as between 2020 to 2022. Google Trends is a popular tool that provides time-series index data queried by users from different given areas. A RSV value represents the relative search frequency and is reported on a scale of 0 to 100.

### Search queries

Table 1 presents the related queries with the most popular search word using Google Trend data. We used search words including “probiotics”, “vitamin B complex”, “vitamin C”, and “vitamin D”. The search period selected was between 2019 to 2022, and the chosen regions included Taiwan and worldwide.

**Table 1:** Related queries with the most popular search words in Taiwan and worldwide between 2019 to 2022

Search Word	Related Queries	
	Taiwan	Worldwide
Probiotics	益生菌 (probiotics)	probiotic
	吃 益生菌(take probiotics)	best probiotics
	推薦 益生菌(probiotics recommendation)	probiotics benefits
		antibiotics
		gut probiotics
		women probiotics
		probiotics supplements
Vitamin B Complex	維他命 b 群 (vitamin B complex)	b complex vitamin benefits
	維他命 b (vitamin B)	vitamin b complex tablet
	推薦 b 群 (vitamin B complex recommendation)	vitamin b12
	b 群 功效 (benefits of vitamin B complex)	b complex uses
	維生素 b (vitamin B)	b vitamins
Vitamin C	維生素 c (vitamin C)	vitamin c tablets
	維生素 c 食物 (food rich in vitamin C)	vitamin c zinc
		vitamin c benefits
	維生素 c 水果 (fruits rich in vitamin C)	best vitamin c
	維生素 c 功效 (benefits of vitamin C)	what is vitamin c
		vitamin c serum
Vitamin D	維他命 d (vitamin D)	vitamin d deficiency
	維生素 d d3 (vitamin D3)	d3 vitamin
	維生素 d 推薦(vitamin D recommendation)	vitamin d foods
	維生素 d 功效(benefits of vitamin D)	vitamin d symptoms
		calcium vitamin d

### Nutritional supplement search interest in different seasons

In our study, we would like to explore whether seasonality may influence the search interest of nutritional supplement both globally and locally. The definitions of seasons used in this study are consistent with other studies and shown below [31]:

- Spring: March 1 to May 31
- Summer: June 1 to August 31
- Autumn: September 1 to November 30
- Winter: December 1 to February 28 (February 29, leap year)

### Feature selection

In this study, Akaike’s Information Criterion (AIC) and stepwise were used for feature selection. AIC is an indicator to measure the goodness-of-fit of the model. A lower AIC means the model fits better. The equation of AIC is shown below:

$$AIC=-2\times \text{l}\text{n}L+2\times k$$

where *L* is the value of the likelihood and *k* is the number of estimated parameters [32].

### Statistical analysis

Statistical analyses were performed by using SAS 9.4 (SAS Institute Inc., Cary, NC, USA.). We analyzed RSVs in web search queries related to four nutritional supplements, including probiotics, vitamin B complex, vitamin C, and vitamin D. Analysis of variance (ANOVA) was used to examine the mean of RSVs in different years and seasons. Tukey’s honest significant difference test used to examine the difference in means between two groups. Spearman’s correlation was used to measure the relationship between RSVs of nutritional supplements and new cases and deaths of COVID-19. Multivariate analysis was conducted to examine the effect of domestic and global new cases and deaths on the RSVs of nutritional supplements. The results show significant differences if *p*-value < 0.05.

## Results

### Relative search volumes (RSVs) for nutritional supplements

**Fig. 3:**
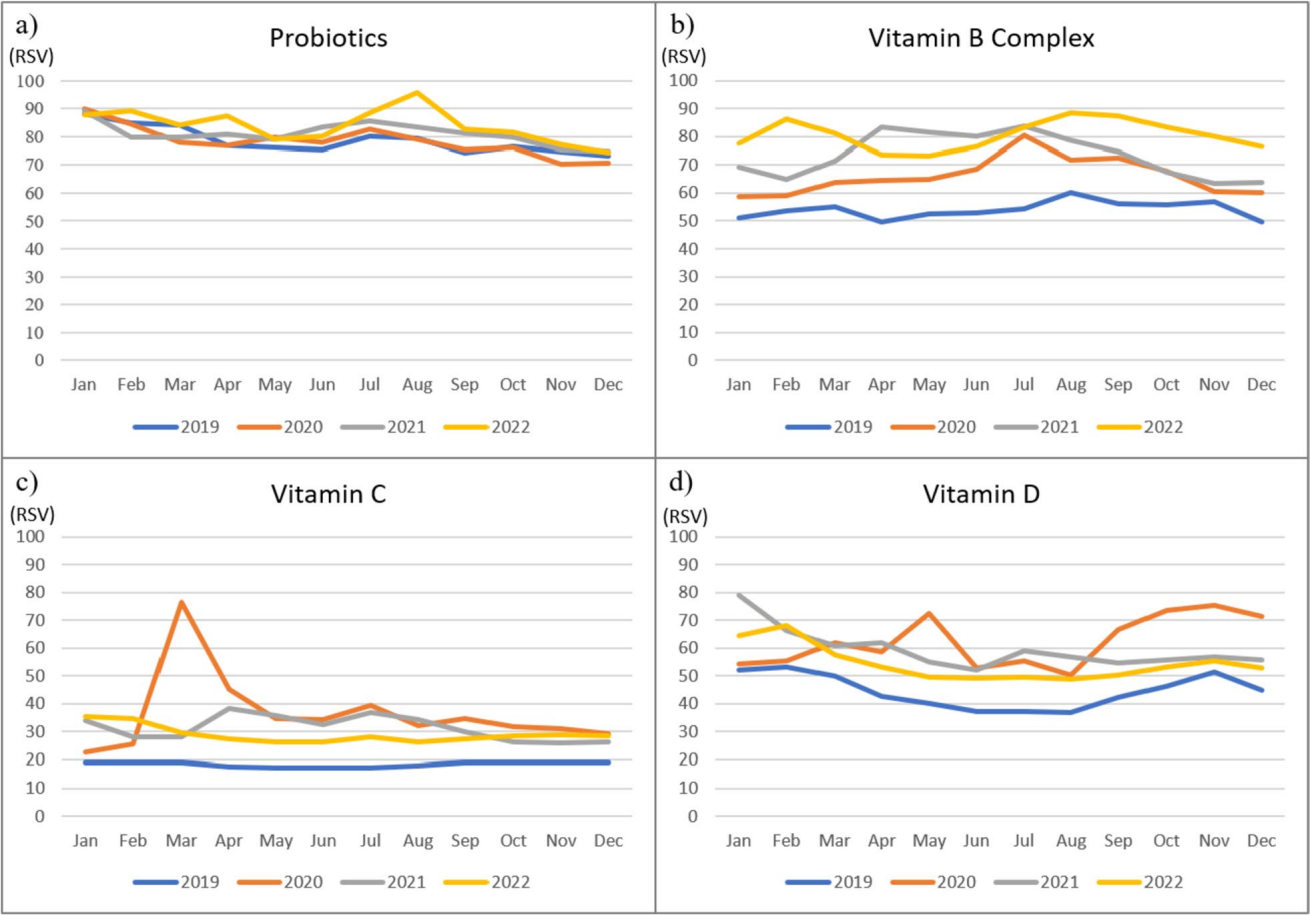
Worldwide monthly mean RSVs for nutritional supplements (Probiotics **A**, Vitamin **B** Complex, Vitamin **C**, and Vitamin **D**) between 2019–2022

We investigated search interests related to nutritional supplements pre- and during the COVID-19 pandemic (Fig. 3). Globally, the mean RSVs for vitamin B complex, vitamin C, and vitamin D worldwide were higher during the COVID-19 pandemic period (between 2020 to 2022) compared to the pre-pandemic period (year of 2019). Interestingly, at the beginning of the COVID-19 outbreak, the RSV for vitamin C peaked in March 2020, suggesting that vitamin C was a popular searched nutritional supplement during the pandemic.

**Fig. 4:**
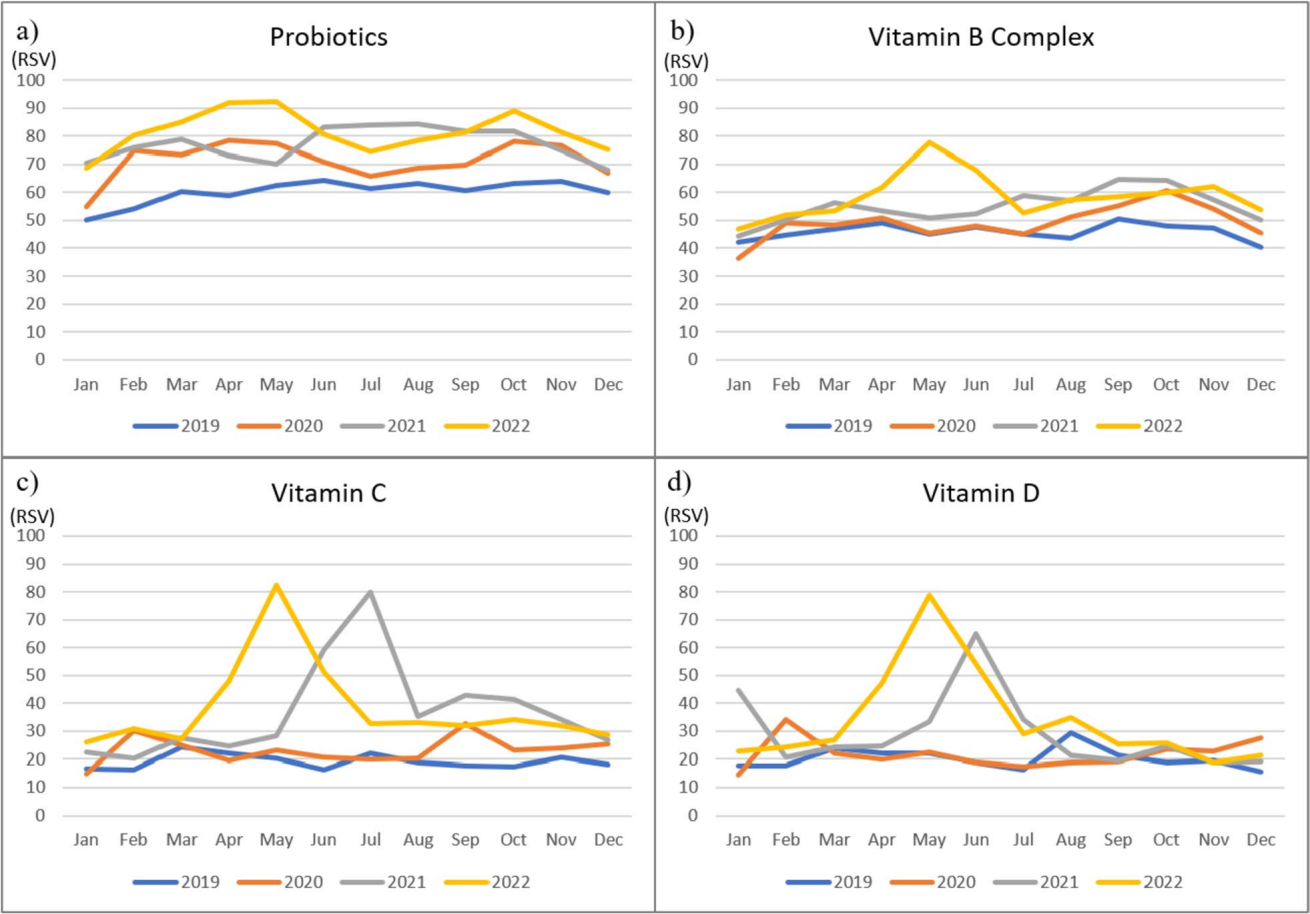
Monthly mean RSVs for nutritional supplements (Probiotics **A**, Vitamin **B** Complex, Vitamin **C**, and Vitamin **D**) between 2019–2022 in Taiwan

The mean RSVs for probiotics, vitamin B, vitamin C, and Vitamin D in Taiwan were also higher during the COVID-19 pandemic period compared to the pre-pandemic period (Fig. 4). RSVs for vitamins C and D increased substantially in May 2021 during the first COVID-19 community transmission in Taiwan. In March 2022, RSVs for vitamin B complex, C, and D increased again due to another COVID-19 (Omicron variant) community transmission.

### Relative search volume (RSVs) for nutritional supplements affected by season and time

**Fig. 5:**
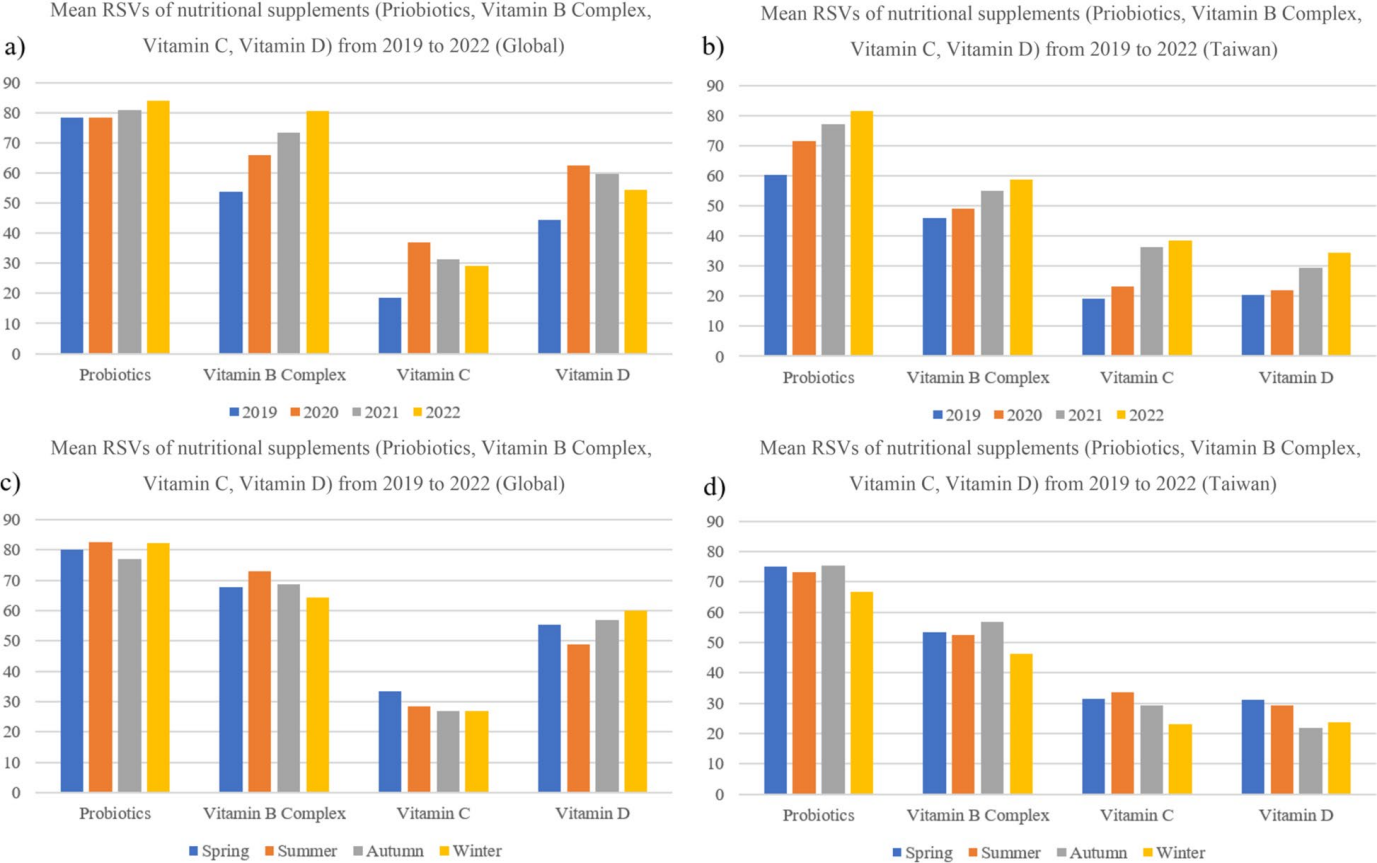
Mean of global and Taiwan RSVs for nutritional supplements from 2019–2022

Moreover, Fig. 5 shows the RSVs for probiotics, vitamin B complex, vitamin C, and vitamin D in Taiwan and worldwide in different seasons and years. As COVID-19 pandemic started in 2020, the search interest for nutritional supplements increased. Figure 5a illustrates that the worldwide RSV for vitamin B complex, vitamin C, and D continuously increased between 2020 to 2022. In Fig. 5b, the RSVs for nutritional supplements increased as well. During the COVID-19 pandemic, the search interest in keywords related to nutritional supplements was increasing every year. Especially for vitamin C and D, the mean RSVs in Taiwan increased significantly while the two community transmissions occurred in 2021 and 2022, respectively. In terms of seasonal variations (Fig. 5d), RSVs showed lower search interest for vitamin D in winter compared to other seasons in Taiwan.

In Taiwan (Tables S3 and S4), RSVs for vitamin B complex, vitamin C, and D increased significantly in 2021, and this could be explained by the first COVID-19 community transmission in Taiwan. Speaking of seasonal variations (Tables S7 and S8), except for vitamin D, the mean RSVs of nutritional supplements in winter were significantly lower than in other seasons. It suggests the RSVs for nutritional supplements could be influenced by seasons. Detailed results of ANOVA and Tukey’s honest significant difference tests can be found in Supplementary Materials (Tables S1 to S8).

### Correlations between RSVs of nutritional supplements and confirmed new COVID-19 cases and deaths

Spearman’s correlation analysis in Table 2 shows that RSV for vitamin B complex had a strong correlation with new COVID-19 cases (*r* = 0.7459), but the search interests for vitamin C and D had higher correlations with new COVID-19 deaths (vitamin C: *r* = 0.7094, vitamin D: *r* = 0.7123). Furthermore, considering seasonal variations, RSVs for vitamin C and D had strong correlations with new COVID-19 cases and deaths in winter.

**Table 2:** Spearman’s correlation coefficients between global RSVs of nutritional supplements and global new cases and deaths

Global RSVs	Global new cases	Global new deaths
Probiotics	0.2703	0.1015
Vitamin B Complex	0.7459	0.5582
Vitamin C	0.5164	0.7094
Vitamin D	0.4807	0.7123
**Spring**		
Probiotics	0.3599	0.0671
Vitamin B Complex	0.9166	0.7809
Vitamin C	0.3365	0.6132
Vitamin D	0.3569	0.6174
**Summer**		
Probiotics	0.6696	0.3677
Vitamin B Complex	0.8333	0.5647
Vitamin C	0.3324	0.8193
Vitamin D	0.4510	0.8104
**Autumn**		
Probiotics	0.1466	0.0367
Vitamin B Complex	0.4297	0.2400
Vitamin C	0.4623	0.6129
Vitamin D	0.4175	0.6730
**Winter**		
Probiotics	0.0860	0.0011
Vitamin B Complex	0.8313	0.6509
Vitamin C	0.8630	0.7729
Vitamin D	0.7054	0.9006

As for Taiwan (Table 3), RSVs of nutritional supplements had higher correlations with local cases and deaths than with global new COVID-19 cases and deaths. In addition, RSVs of nutritional supplements had higher correlations with local cases and deaths in all seasons except winter.

**Table 3:** Spearman’s correlation coefficients between Taiwan’s RSVs of nutritional supplements, global new cases and deaths, and local new cases and deaths

Taiwan’s RSVs	Global new cases	Global new deaths	Local new cases	Local new deaths
Probiotics	0.5796	0.4749	0.7289	0.5649
Vitamin B complex	0.4032	0.2462	0.5667	0.5059
Vitamin C	0.5071	0.3110	0.6806	0.6052
Vitamin D	0.3304	0.2216	0.4103	0.3531
**Spring**				
Probiotics	0.6632	0.3528	0.7117	0.4341
Vitamin B complex	0.5192	0.2283	0.5743	0.4139
Vitamin C	0.4133	0.0484	0.5818	0.5068
Vitamin D	0.3204	0.0625	0.4745	0.3798
**Summer**				
Probiotics	0.7242	0.6237	0.7391	0.7255
Vitamin B complex	0.6345	0.2789	0.6813	0.6785
Vitamin C	0.6738	0.4931	0.7772	0.7787
Vitamin D	0.4061	0.1281	0.5964	0.6836
**Autumn**				
Probiotics	0.5990	0.4680	0.8081	0.6832
Vitamin B complex	0.3628	0.4037	0.5477	0.4503
Vitamin C	0.4233	0.4506	0.5731	0.4323
Vitamin D	0.1493	0.0972	0.2087	0.1636
**Winter**				
Probiotics	0.5584	0.5786	0.6219	0.2783
Vitamin B complex	0.3540	0.2871	0.5120	0.2722
Vitamin C	0.5157	0.3243	0.6095	0.3966
Vitamin D	0.4201	0.5236	0.3615	0.2017

### Multivariate regression analysis and lag effect

Lag 0, -1, -2, and − 3 week were incorporated in the multivariate regression model. During the process of feature selection, global new case, global new case Lag − 3 and death Lag-3 were not selected as predictors. In the regression analysis (Table 4), we observed RSVs of nutritional supplements were not only influenced by new cases and deaths at the present time but also by the time before the presence of new cases and deaths.

**Table 4:** Multivariate analysis for global RSVs of nutritional supplements via feature selection

Global RSVs	β	Standard Error	*p*-value
**Probiotics**			
Global new death	-0.000034	0.000020	0.0822
Global new case_lag2	0.000001	0.000000	< 0.0001***
**Vitamin B Complex**			
Global new death	-0.000069	0.000025	0.0071**
Global new case_lag2	0.000001	0.000000	< 0.0001***
**Vitamin C**			
Global new death	0.000356	0.000093	0.0002***
Global new case_lag1	0.000000	0.000000	0.0246*
Global new death_lag2	-0.000369	0.000092	< 0.0001***
**Vitamin D**			
Global new death	0.00053615	0.000155	0.0007***
Global new death_lag1	-0.00055613	0.000155	0.0004***

*, *p* < 0.05 **, *p* < 0.01; ***, *p* < 0.001

In Taiwan, we assumed that RSVs of nutritional supplements were not only affected by local COVID-19 new cases and deaths but also by global new cases and deaths. Our results showed RSVs of nutritional supplements were affected by the local COVID-19 new cases and deaths, including Lag 0, Lag − 1, and Lag − 2, as well as affected by Lag − 3 of global new COVID-19 cases and deaths (Table 5).

**Table 5:** Multivariate analysis for RSVs of nutritional supplements in Taiwan via feature selection

	β	Standard Error	*P*-value
**Probiotics**			
Local new case	0.000038	0.000010	0.0003***
Local new case _lag2	-0.000024	0.000010	0.0210*
Global new case_lag1	-0.000001	0.000000	0.0011**
Global new death_lag1	-0.000220	0.000071	0.0022**
Global new case_lag3	0.000001	0.000000	< 0.0001***
Global new death_lag3	0.000184	0.000069	0.0087**
**Vitamin B Complex**			
Local new case _lag1	0.000111	0.000017	< 0.0001***
Local new case _lag2	-0.000081	0.000017	< 0.0001***
Global new case_lag1	-0.000001	0.000001	0.0525
Global new case_lag2	0.000001	0.000001	0.0594
Global new death_lag2	-0.000385	0.000117	0.0012**
Global new death_lag3	0.000370	0.000115	0.0016**
**Vitamin C**			
Local new case	0.000167	0.000036	< 0.0001***
Local new death	0.095820	0.022820	< 0.0001***
Local new case _lag1	-0.000084	0.000057	0.1442
Local new case _lag2	-0.000148	0.000061	0.0163*
Local new case _lag3	-0.000056	0.000040	0.1682
Global new death_lag2	-0.000795	0.000214	0.0003***
Global new death_lag3	0.000825	0.000211	0.0001***
**Vitamin D**			
Local new case	0.000160	0.000039	< 0.0001***
Local new death	0.112060	0.024100	< 0.0001***
Local new case _lag1	-0.000127	0.000062	0.0429*
Local new case _lag2	-0.000105	0.000066	0.1167
Local new case _lag3	-0.000082	0.000042	0.0556
Global new case_lag2	-0.000003	0.000001	0.0129*
Global new case_lag3	0.000003	0.000001	0.0053**

*, *p* < 0.05 **, *p* < 0.01; ***, *p* < 0.001

The RSVs of nutritional supplements in Taiwan were not influenced by global new cases and deaths and local new deaths in the time before the presence of new cases and deaths. Our results showed the RSV of vitamin B complex was only affected by the lag effect. We also observed RSVs of vitamin B complex, vitamin C, vitamin D, and probiotics were not only affected by local new cases and deaths but also by global new cases and deaths.

## Discussion

In this study, we aim to compare the public interest in nutritional supplements pre- and during the COVID-19 pandemic in Taiwan and worldwide, as well as determining the potential factors which affect search interest of nutritional supplements. The mean RSVs for nutritional supplements were higher during the COVID-19 pandemic period compare to the pre-pandemic period for both Taiwan and worldwide. Speaking of seasonal variation, except for vitamin D, the mean RSVs of probiotics, vitamin B complex, and vitamin C in winter were significantly lower compared to other seasons in Taiwan. Overall, the RSVs of nutritional supplements appeared to not only be influenced by local pandemic severity but also by global new cases and deaths associated with COVID-19.

The first COVID-19 case in Taiwan was reported on January 21st, 2020, and later the World Health Organization declared a pandemic on March 11, 2020. Our present study showed the search on vitamins and probiotics around the world increased rapidly in March 2020, and all search RSVs for vitamins and probiotics continue to rise between 2021 and 2022. A couple of significant events may contribute to the high search interest for vitamins and probiotics. First, the death toll from COVID-19 exceeded 500,000 in the USA in February 2021. Same year in April, the global tally of deaths from COVID-19 surpassed 3 million. Later in the summer, WHO announced that a more dangerous and the most transmissible SARS-CoV-2 virus to date, named Delta variant, was discovered in August. In November, WHO discovered another new coronavirus variant Omicron, and the Omicron variant had been reported in 89 countries [4]. In comparison, our study found the search for vitamins and probiotics in Taiwan increased in later years (2021–2022). In addition, there were sudden peaks of RSVs for vitamin C and D during the years of 2021–2022. The sudden peaks of RSVs for vitamin C and D could be explained by the local community outbreak of COVID-19 in May 2021 in Taiwan [33]. All the above observations showed that interest in nutritional supplementation for non-Taiwanese individuals had risen substantially during the peaks of the global COVID-19 pandemic. By contrast, the Taiwanese’s interest in nutritional supplementation was influenced primarily by the local pandemic.

Nutritional supplements with antimicrobial and immunomodulatory activity are known to be effective in prevention of viral spreading and therapeutic adjuvants for the treatment of COVID-19 [34, 35]. Between 2021 and 2022 while Taiwan was facing escalating COVID-19 cases and deaths, individuals started looking into nutritional supplementation in the hope of preventing COVID-19 as well as reducing symptoms severity. According to our study findings, the search volumes for nutritional supplements including probiotics, vitamin B complex, vitamin C, and vitamin D increased, with vitamin C and D search volumes having sudden peaks during the two local community outbreaks between 2021 and 2022. A study by Cheng and colleagues suggested that early and high intravenous doses of vitamin C injection may be helpful in preventing and treating coronavirus infection [35]. Vitamin D is an important anti-inflammatory nutrient, and a research by Grant and colleagues proposed that vitamin D may reduce the risk of coronavirus infection by decreasing the viral replication rate through the host defense system [36]. A similar non-pharmaceutical approach to prevent COVID-19 was also observed in other countries as well. In Sri Lanka, A study by Francis and colleagues conducted an online survey to investigate the frequency and composition of nutritional supplements consumed by Sri Lankans. The study showed a larger proportion of Sri Lankans used Vitamin C supplements during the COVID-19 pandemic due to its antimicrobial and immunomodulatory properties [37]. Similarly, other population-based PLifeCOVID-19 Studies also showed consistent results – Vitamin C and vitamin D were the most supplemented compounds since the beginning of the COVID-19 pandemic [12].

We found that interest in vitamins and probiotics showed seasonality in Taiwan. Globally, the search popularity for vitamin B Complex, Vitamin C, Vitamin D, and probiotics didn’t vary much among different seasons. This appears to be a new phenomenon as in the past, the interest in nutritional supplement use was usually the lowest during June, July, and August as these are the months with warm weather in the northern hemisphere [30]. This may be partially explained by the absence of urgent needs (i.e. vitamin D due to insufficient daylight exposure, vitamin C for common colds) for nutritional supplements [30]. Nevertheless, during the major COVID-19 pandemic period (2019–2022), nutritional supplement uses were in demand in all seasons as people from different parts of the world were frequently seeking nonpharmaceutical options to enhance their immune systems in face of the global COVID-19 pandemic [38]. In Taiwan between 2019 and 2022, the interest in vitamin B complex appeared to be higher in autumn, while the interest in vitamin C and D appeared to be higher in spring and summer, and the interest in probiotics appeared to be higher in spring and autumn. People are more likely to engage in outdoor activities during spring, summer, and fall, and supplementation of nutritional supplements could help in improving activity performance, enhancing immunity, and reducing the severity of allergy symptoms [39].

Furthermore, it was observed from our study that the mean RSV of nutritional supplements increased notably after the announcement of local pandemic outbreaks. The reported number of COVID-19 cases in Taiwan surged in March (spring) 2020 and in May & June (spring & summer) in 2021 & 2022, this may explain why the interest in vitamin B complex, vitamin C, vitamin D, and probiotics was also higher in these seasons [33].

Our study found that the relative search volume of nutritional supplements was influenced by new COVID-19 cases and deaths. Globally, around 3.4 billion people have access to the internet and online information has become the source for many people to seek health information [40, 41]. About 55% of American adults have used the internet to get information related to health or illness, called “health seekers”. 70% of health seekers’ decisions on treating an illness were influenced by online information. For illness, 13% have sought information related to nutrition [42]. A study by Mayasari and colleagues pointed out that the pandemic has a profound impact on lifestyle behaviors by using Google Trends covering a timeframe from 1 June 2019 to 27 April 2020. Compared to the period before the COVID-19 pandemic, RSVs of vitamins, particularly vitamin C, increased significantly after the pandemic outbreak [27]. Kushwaha and colleagues also found that daily new COVID-19 cases in India were highly correlated to search terms related to immune-related nutrients, including “multivitamin”, “nutrients”, and “vitamin” in different time lag days by using cross-correlation analysis [43]. In our results, the interests in nutritional supplements were influenced by the time before new COVID-19 cases and deaths occurred. Therefore, we can conclude that with the changes in the pandemic situation, Taiwanese online searching behaviors for nutritional supplements will change as well.

There were several limitations that are important to consider. First, the study uses English search terms and Google as the search engine, which may not accurately reflect the global interests. Second, the relative search volumes in Google Trends were only relative volumes and not absolute values. Third, using Our World in Data to reflect a local situation may have a potential reliability concern due to incomplete reporting compare to using data retrieved from local government database. Fourth, this is a retrospective study where we can not guarantee the search interests of nutritional supplements remain unchanged during the COVID-19 pandemic. The strengths of this study included the use of worldwide RSV data and comprehensive search terms related to nutritional supplements. The surveillance of online search queries can offer insights into differences of interest in nutritional supplements among different populations (Taiwan vs. worldwide) in response to the COVID-19 pandemic.

## Conclusion

Our study showed that interests in nutritional supplements are affected by global pandemic situation as well as local pandemic outbreaks. The relative search volumes of nutritional supplements in Taiwan were mainly influenced by local new COVID-19 cases and deaths. There is also a seasonal variation in the interest of nutritional supplements in Taiwan. Currently, there is no clear evidence to support the role of nutritional supplements in COVID-19 prevention and the potential risk of elevated intake of some nutrients due to the popularity of nutritional supplements, effective education on the rationale of nutritional supplement use should be developed at local and even national levels.

### Supplementary Information

**Additional file 1: Table S1.** Comparison of global GRSVs of mean between the four years by ANOVA test. **Table S2.** Comparison of global GRSVs of mean between the four years by Tukey HSD test. **Table S3.** Comparison of Taiwan GRSVs of mean between the four years by ANOVA test. **Table S4.** Comparison of Taiwan GRSVs of mean between the four years by Tukey HSD test. **Table S5.** Comparison of global GRSVs of mean between the four seasons by ANOVA test. **Table S6.** Comparison of global GRSVs of mean between the four seasons by Tukey HSD test. **Table S7.** Comparison of Taiwan GRSVs of mean between the four season by  ANOVA test. **Table S8.** Comparison of Taiwan GRSVs of mean between the four seasons by  Tukey HSD test.

## Data Availability

The datasets used and/or analyzed during the current study are available for download at the following websites: https://ourworldindata.org/coronavirus and https://trends.google.com.tw/trends/.
