# Supplementary material for: The association between interest of nutritional supplements and COVID-19 pandemic - evidence from Google Trends
Source: BMC Public Health. 2024 Jan 6;24:109. doi: 10.1186/s12889-023-17607-2 (PMC10771707; doi:10.1186/s12889-023-17607-2)
Supplement: Supplementary file 1 — Additional file 1: Table S1. Comparison of global GRSVs of mean between the four years by ANOVA test. Table S2. Comparison of global GRSVs of mean between the four years by Tukey HSD test. Table S3. Comparison of Taiwan GRSVs of mean between the four years by ANOVA test. Table S4. Comparison of Taiwan GRSVs of mean between the four years by Tukey HSD test. Table S5. Comparison of global GRSVs of mean between the four seasons by ANOVA test. Table S6. Comparison of global GRSVs of mean between the four seasons by Tukey HSD test. Table S7. Comparison of Taiwan GRSVs of mean between the four season by ANOVA test. Table S8. Comparison of Taiwan GRSVs of mean between the four seasons by Tukey HSD test. [file 12889_2023_17607_MOESM1_ESM.docx]

**Supplementary Tables**

**Table S1 Comparison of global GRSVs of mean between the four years by ANOVA test**

|  | Df | Sum Sq | Mean Sq | F value | Pr(>F) |
| --- | --- | --- | --- | --- | --- |
| Probiotics |  |  |  |  |  |
| Year | 3 | 1130.21 | 376.74 | 9.52 | <.0001*** |
| Residuals | 204 | 8073.67 | 39.58 |  |  |
| Vitamin B Complex |  |  |  |  |  |
| Year | 3 | 20274.73 | 6758.24 | 159.55 | <.0001*** |
| Residuals | 204 | 8641.19 | 42.36 |  |  |
| Vitamin C |  |  |  |  |  |
| Year | 3 | 9386.36 | 3128.79 | 45.33 | <.0001*** |
| Residuals | 204 | 14080.40 | 69.02 |  |  |
| Vitamin D |  |  |  |  |  |
| Year | 3 | 9889.75 | 3296.58 | 50.77 | <.0001*** |
| Residuals | 204 | 13246.63 | 64.93 |  |  |

**Table S2 Comparison of global GRSVs of mean between the four years by Tukey HSD test**

|  | The difference of mean | 95% C.I. | | *p*-value |
| --- | --- | --- | --- | --- |
| Probiotic | | | | |
| 2019 - 2020 | 0.173 | -3.023 | 3.369 |  |
| 2019 - 2021 | -2.596 | -5.792 | 0.6 |  |
| 2019 - 2022 | -5.558 | -8.754 | -2.362 | *** |
| 2020 - 2021 | -2.769 | -5.965 | 0.427 |  |
| 2020 - 2022 | -5.731 | -8.927 | -2.535 | *** |
| 2021 - 2022 | -2.962 | -6.157 | 0.234 |  |
| Vitamin B complex | | | | |
| 2019 - 2020 | -12.038 | -15.345 | -8.732 | *** |
| 2019 - 2021 | -19.635 | -22.941 | -16.328 | *** |
| 2019 - 2022 | -26.635 | -29.941 | -23.328 | *** |
| 2020 - 2021 | -7.596 | -10.902 | -4.29 | *** |
| 2020 - 2022 | -14.596 | -17.902 | -11.29 | *** |
| 2021 - 2022 | -7 | -10.306 | -3.694 | *** |
| Vitamin C | | | | |
| 2019 - 2020 | -18.5 | -22.72 | -14.28 | *** |
| 2019 - 2021 | -13 | -17.22 | -8.78 | *** |
| 2019 - 2022 | -10.596 | -14.817 | -6.376 | *** |
| 2020 - 2021 | 5.5 | 1.28 | 9.72 | *** |
| 2020 - 2022 | 7.904 | 3.683 | 12.124 | *** |
| 2021 - 2022 | 2.404 | -1.817 | 6.624 |  |
| Vitamin D | | | | |
| 2019 - 2020 | -18.115 | -22.209 | -14.022 | *** |
| 2019 - 2021 | -15.173 | -19.267 | -11.079 | *** |
| 2019 - 2022 | -9.846 | -13.94 | -5.753 | *** |
| 2020 - 2021 | 2.942 | -1.151 | 7.036 |  |
| 2020 - 2022 | 8.269 | 4.176 | 12.363 | *** |
| 2021 - 2022 | 5.327 | 1.233 | 9.421 | *** |

**Table S3 Comparison of Taiwan GRSVs of mean between the four years by ANOVA test**

|  | Df | Sum Sq | Mean Sq | F value | Pr(>F) |
| --- | --- | --- | --- | --- | --- |
| Probiotics |  |  |  |  |  |
| Year | 3 | 13161.90 | 4387.30 | 78.97 | <.0001*** |
| Residuals | 204 | 11333.77 | 55.56 |  |  |
| Vitamin B Complex |  |  |  |  |  |
| Year | 3 | 5136.33 | 1712.11 | 26.47 | <.0001*** |
| Residuals | 204 | 13196.11 | 64.69 |  |  |
| Vitamin C |  |  |  |  |  |
| Year | 3 | 14308.42 | 4769.47 | 25.77 | <.0001*** |
| Residuals | 204 | 37762.81 | 185.112 |  |  |
| Vitamin D |  |  |  |  |  |
| Year | 3 | 6893.25 | 2297.75 | 11.05 | <.0001*** |
| Residuals | 204 | 42424.75 | 207.96 |  |  |

**Table S4 Comparison of Taiwan GRSVs of mean between the four years by**

**Tukey HSD test**

|  | The difference of mean | 95% C.I. | | *p*-value |
| --- | --- | --- | --- | --- |
| Probiotic | | | | |
| 2019 - 2020 | -11.269 | -15.056 | -7.483 | *** |
| 2019 - 2021 | -16.808 | -20.594 | -13.021 | *** |
| 2019 - 2022 | -21.269 | -25.056 | -17.483 | *** |
| 2020 - 2021 | -5.538 | -9.325 | -1.752 | *** |
| 2020 - 2022 | -10 | -13.787 | -6.213 | *** |
| 2021 - 2022 | -4.462 | -8.248 | -0.675 | *** |
| Vitamin B complex | | | | |
| 2019 - 2020 | -3.327 | -7.413 | 0.759 |  |
| 2019 - 2021 | -9.019 | -13.105 | -4.933 | *** |
| 2019 - 2022 | -12.846 | -16.932 | -8.76 | *** |
| 2020 - 2021 | -5.692 | -9.778 | -1.607 | *** |
| 2020 - 2022 | -9.519 | -13.605 | -5.433 | *** |
| 2021 - 2022 | -3.827 | -7.913 | 0.259 |  |
| Vitamin C | | | | |
| 2019 - 2020 | -4.038 | -10.95 | 2.873 |  |
| 2019 - 2021 | -17.212 | -24.123 | -10.3 | *** |
| 2019 - 2022 | -19.365 | -26.277 | -12.454 | *** |
| 2020 - 2021 | -13.173 | -20.085 | -6.261 | *** |
| 2020 - 2022 | -15.327 | -22.239 | -8.415 | *** |
| 2021 - 2022 | -2.154 | -9.066 | 4.758 |  |
| Vitamin D | | | | |
| 2019 - 2020 | -1.558 | -8.884 | 5.768 |  |
| 2019 - 2021 | -9.096 | -16.422 | -1.77 | *** |
| 2019 - 2022 | -14.212 | -21.537 | -6.886 | *** |
| 2020 - 2021 | -7.538 | -14.864 | -0.213 | *** |
| 2020 - 2022 | -12.654 | -19.98 | -5.328 | *** |
| 2021 - 2022 | -5.115 | -12.441 | 2.211 |  |

**Table S5 Comparison of global GRSVs of mean between the four seasons by ANOVA test**

|  | Df | Sum Sq | Mean Sq | F value | Pr(>F) |
| --- | --- | --- | --- | --- | --- |
| Probiotics |  |  |  |  |  |
| Season | 3 | 1001.15 | 333.72 | 8.30 | <.0001*** |
| Residuals | 204 | 8202.73 | 40.21 |  |  |
| Vitamin B Complex |  |  |  |  |  |
| Season | 3 | 2032.40 | 677.47 | 5.14 | 0.0019** |
| Residuals | 204 | 26883.52 | 131.78 |  |  |
| Vitamin C |  |  |  |  |  |
| Season | 3 | 1531.90 | 510.63 | 4.75 | 0.0032** |
| Residuals | 204 | 21934.86 | 107.52 |  |  |
| Vitamin D |  |  |  |  |  |
| Season | 3 | 3432.41 | 1144.14 | 11.85 | <.0001*** |
| Residuals | 204 | 19703.97 | 96.59 |  |  |

**Table S6 Comparison of global GRSVs of mean between the four seasons by Tukey HSD test**

|  | The difference of mean | 95% C.I. | | p-value |
| --- | --- | --- | --- | --- |
| Probiotic | | | | |
| spring - summer | -2.445 | -5.651 | 0.761 |  |
| spring - autumn | 3.094 | -0.112 | 6.3 |  |
| spring - winter | -2.064 | -5.286 | 1.158 |  |
| summer - autumn | 5.538 | 2.317 | 8.76 | *** |
| summer - winter | 0.381 | -2.856 | 3.618 |  |
| autumn - winter | -5.158 | -8.395 | -1.921 | *** |
| Vitamin B complex | | | | |
| spring - summer | -5.247 | -11.051 | 0.557 |  |
| spring - autumn | -0.786 | -6.59 | 5.019 |  |
| spring - winter | 3.575 | -2.258 | 9.407 |  |
| summer - autumn | 4.462 | -1.37 | 10.293 |  |
| summer - winter | 8.822 | 2.961 | 14.682 | *** |
| autumn - winter | 4.36 | -1.5 | 10.22 |  |
| Vitamin C | | | | |
| spring - summer | 4.991 | -0.252 | 10.234 |  |
| spring - autumn | 6.683 | 1.44 | 11.926 | *** |
| spring - winter | 6.472 | 1.203 | 11.74 | *** |
| summer - autumn | 1.692 | -3.575 | 6.96 |  |
| summer - winter | 1.481 | -3.813 | 6.774 |  |
| autumn - winter | -0.212 | -5.505 | 5.082 |  |
| Vitamin D | | | | |
| spring - summer | 6.721 | 1.752 | 11.69 | *** |
| spring - autumn | -1.298 | -6.267 | 3.671 |  |
| spring - winter | -4.451 | -9.444 | 0.543 |  |
| summer - autumn | -8.019 | -13.012 | -3.027 | *** |
| summer - winter | -11.172 | -16.189 | -6.155 | *** |
| autumn - winter | -3.153 | -8.17 | 1.864 |  |

**Table S7 Comparison of Taiwan GRSVs of mean between the four season by ANOVA test**

|  | Df | Sum Sq | Mean Sq | F value | Pr(>F) |
| --- | --- | --- | --- | --- | --- |
| Probiotics |  |  |  |  |  |
| Season | 3 | 2603.35 | 867.78 | 8.09 | <.0001*** |
| Residuals | 204 | 21892.30 | 107.32 |  |  |
| Vitamin B Complex |  |  |  |  |  |
| Season | 3 | 3090.90 | 1030.30 | 13.79 | <.0001*** |
| Residuals | 204 | 15241.54 | 74.71 |  |  |
| Vitamin C |  |  |  |  |  |
| Season | 3 | 3228.70 | 1076.23 | 4.50 | 0.0044** |
| Residuals | 204 | 48842.54 | 239.42 |  |  |
| Vitamin D |  |  |  |  |  |
| Season | 3 | 3165.41 | 1055.14 | 4.66 | 0.0036** |
| Residuals | 204 | 46152.59 | 226.24 |  |  |

**Table S8 Comparison of Taiwan GRSVs of mean between the four seasons by Tukey HSD test**

|  | The difference of mean | 95% C.I. | | *p*-value |
| --- | --- | --- | --- | --- |
| Probiotic | | | | |
| spring - summer | 1.864 | -3.374 | 7.101 |  |
| spring - autumn | -0.271 | -5.509 | 4.967 |  |
| spring - winter | 8.526 | 3.262 | 13.789 | *** |
| summer - autumn | -2.135 | -7.397 | 3.128 |  |
| summer - winter | 6.662 | 1.374 | 11.95 | *** |
| autumn - winter | 8.797 | 3.508 | 14.085 | *** |
| Vitamin B complex | | | | |
| spring - summer | 0.976 | -3.395 | 5.346 |  |
| spring - autumn | -3.601 | -7.972 | 0.769 |  |
| spring - winter | 7.166 | 2.774 | 11.558 | *** |
| summer - autumn | -4.577 | -8.968 | -0.186 | *** |
| summer - winter | 6.19 | 1.778 | 10.603 | *** |
| autumn - winter | 10.767 | 6.355 | 15.18 | *** |
| Vitamin C | | | | |
| spring - summer | -1.972 | -9.795 | 5.851 |  |
| spring - autumn | 2.22 | -5.603 | 10.044 |  |
| spring - winter | 8.567 | 0.705 | 16.429 | *** |
| summer - autumn | 4.192 | -3.668 | 12.053 |  |
| summer - winter | 10.539 | 2.64 | 18.438 | *** |
| autumn - winter | 6.347 | -1.552 | 14.246 |  |
| Vitamin D | | | | |
| spring - summer | 1.843 | -5.762 | 9.448 |  |
| spring - autumn | 9.42 | 1.815 | 17.025 | *** |
| spring - winter | 7.542 | -0.1 | 15.185 |  |
| summer - autumn | 7.577 | -0.064 | 15.218 |  |
| summer - winter | 5.699 | -1.979 | 13.378 |  |
| autumn - winter | -1.877 | -9.556 | 5.801 |  |
